# 15-Month Health Outcomes and the Related Risk Factors of Hospitalized COVID-19 Patients From Onset: A Cohort Study

**DOI:** 10.3389/fmed.2022.854788

**Published:** 2022-05-11

**Authors:** Liang-Liang Sun, Jian Wang, Yu-Sheng Wang, Xiao Pan, Jun Luo, Hua Liu, Yi-Rou Jiang, Xin Zhuang, Liang Lin, Gan-Cheng Li, Jun-Wei Zhao, Wei Wang, Yuan-Jing Wang, Zhi-Hao Wang, Hong-Biao Shan, Shuai-Shuai Chen, Jun-Lin Chen, Zhao-Wei Xu, Yong-Hai Bai, Hai Huang, Wei-Fen Xie

**Affiliations:** ^1^Department of Endocrinology and Metabolism, Changzheng Hospital, Naval Medical University, Shanghai, China; ^2^Department of Gastroenterology, Changzheng Hospital, Naval Medical University, Shanghai, China; ^3^Department of Respiratory and Critical Care Medicine, Changzheng Hospital, Naval Medical University, Shanghai, China; ^4^Department of Medical Psychology, Changzheng Hospital, Naval Medical University, Shanghai, China; ^5^Department of General Practice, Changzheng Hospital, Naval Medical University, Shanghai, China

**Keywords:** long-term health consequence, COVID-19, persistent symptom, mental health, PTSD

## Abstract

**Objective:**

The long-term impact of COVID-19 on patient health has been a recent focus. This study aims to determine the persistent symptoms and psychological conditions of patients hospitalized with COVID-19 15 months after onset, that patients first developed symptoms. The potential risk factors were also explored.

**Methods:**

A cohort of COVID-19 patients discharged from February 20, 2020 to March 31, 2020 was recruited. Follow-ups were conducted using validated questionnaires and psychological screening scales at 15 months after onset to evaluate the patients' health status. The risk factors for long-term health impacts and their associations with disease severity was analyzed.

**Findings:**

534 COVID-19 patients were enrolled. The median age of the patients was 62.0 years old (IQR 52.0–70.0) and 295 were female (55.2%). The median time from onset to follow-up was 460.0 (451.0–467.0) days. Sleep disturbance (18.5%, 99/534) and fatigue (17.2%, 92/534) were the most common persistent symptoms. 6.4% (34/534) of the patients had depression, 9.2% (49/534) were anxious, 13.0% (70/534) had insomnia and 4.7% (25/534) suffered from post-traumatic stress disorder (PTSD). Multivariate adjusted logistic regression analysis showed that glucocorticoid use during hospitalization (OR 3.58, 95% CI 1.12–11.44) was significantly associated with an increased risk of fatigue. The OR values for anxiety and sleep disorders were 2.36 (95% CI 1.07–5.20) and 2.16 (95% CI 1.13–4.14) in females to males. The OR value of PTSD was 25.6 (95% CI 3.3–198.4) in patients with persistent symptoms to those without persistent symptoms. No significant associations were observed between fatigue syndrome or adverse mental outcomes and disease severity.

**Conclusions:**

15-month follow-up in this study demonstrated the need of extended rehabilitation intervention for complete recovery in COVID-19 patients.

## Introduction

Severe acute respiratory syndrome coronavirus 2 (SARS-CoV-2) is the pathogen responsible for the coronavirus disease 2019 (COVID-19) pandemic, which has resulted in global healthcare crises and strained health resources (1). Globally, as of 12 March 2022, there have been 452,201,564 confirmed cases of COVID-19, including 6,029,852 deaths, reported to WHO (2).

COVID-19-related symptoms have been intensively studied in different systems since the pandemic outbreak. Most COVID-19 patients suffer from respiratory symptoms (such as fever, cough, and dyspnea) and are subjected to multiple organ injuries caused by SARS-CoV-2 infection together with the drugs used in the treatment of this disease (3, 4). Currently, researchers are aware of the persistent symptoms of COVID-19 after recovery, which are defined as “post-COVID condition,” “long COVID” or “post-COVID syndrome,” indicating a long-term course of various physical and neuropsychiatric symptoms lasting more than 12 weeks without other explanation (5, 6).

Long COVID is a rapidly evolving medical problem that requires action now. Several recent studies have reported specific persistent symptoms in discharged patients, such as fatigue and dyspnea (6). The severity of this disease in acute phase is likely to be related to the long-term adverse outcome of the disease, and gender may be an important risk factor affecting the adverse psychological outcome (7). However, to date, most studies have only examined adverse health effects up to 6 months after Covid-19 diagnosis, and little is known about the long-term mental health effects. It is still unclear how long COVID lasts, what the risk factors for long COVID are, and the relationship between long COVID and disease severity during the acute phase. Therefore, there is an urgent need to clearly define the long-term impact of COVID-19 on health in recovered patients and its potential risk factors.

Recently, we conducted a research to describe the detailed symptomatic features of COVID-19 at the onset and rehabilitation stages (8). The data showed that COVID-19 patients presented atypical but diverse symptoms. The most common remaining symptoms at the 3-month recovery stage were cough and fatigue. The proportion and severity of dyspnea as a remaining symptom after discharge in severe patients were higher than those in non-severe patients.

In this study, we aimed to explore the clinical characteristics of long COVID and especially to discuss the remaining long-term mental and psychological problems and their related risk factors. This study provide an important and critical update to our previously published data on the symptomatic characteristics and prognosis of COVID-19 (8).

## Methods

### Study Design and Participants

All the patients enrolled in this study were from the same cohort in our other recently published study (8). The subjects included in our cohort were diagnosed with COVID-19 by reverse transcription-polymerase chain reaction (RT–PCR) and were discharged from the Optical Valley Branch of Hubei Maternal and Child Hospital, a designated hospital for COVID-19 patients in Wuhan, from February 20 to March 31, 2020.

The following patients were excluded: (1) patients who died after discharge; (2) patients who were difficult to follow up due to mental illness, dementia, or underlying diseases; (3) patients who refused to cooperate; (4) patients who could not be contacted; and (5) patients who lived in nursing homes or welfare homes. All the study participants provided informed consent. The Research Ethics Committee of Shanghai Changzheng Hospital approved this study (2020SL007).

**Figure 1:**
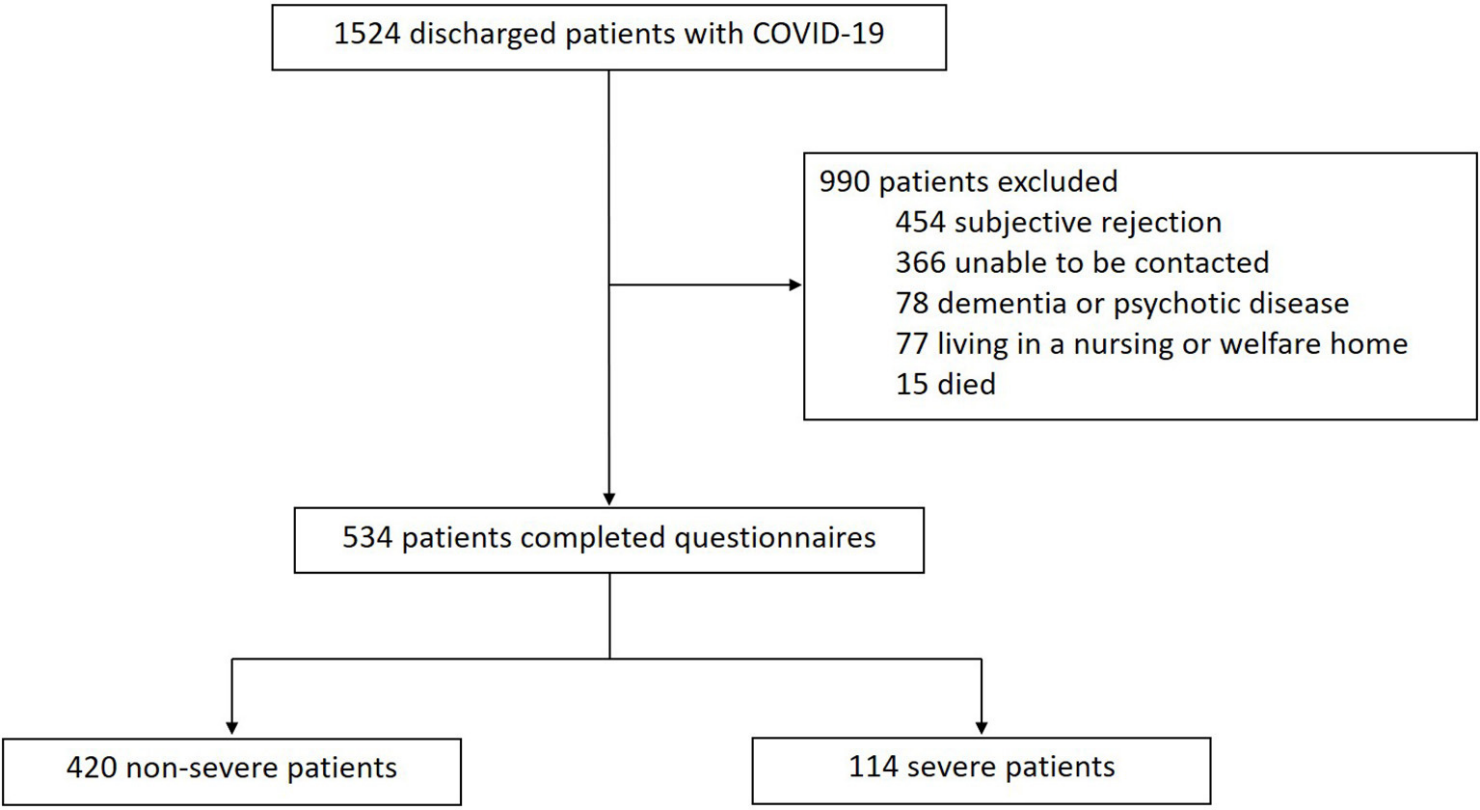
Flow chart of hospital patients with COVID-19.

A total of 1,524 patients with COVID-19 discharged from the Guanggu District of Hubei Maternal and Child Healthcare Hospital between February 20 and March 31, 2020 were screened. As shown in Figure 1, 990 patients were excluded, of which 454 refused to cooperate, 366 could not be contacted, 78 had dementia or psychotic disease who could not complete the interview, 77 lived in nursing or welfare home, and 15 died. Lastly, 534 patients were enrolled in this study, including 114 severe cases and 420 non-severe cases.

### Procedures

The collected data of acute phase were extracted from electronic medical records of patients with COVID-19 hospitalized in Optical Valley Branch of Maternal and Child Hospital of Hubei Province, including demographic information and clinical characteristics, which were described in our previous study (8). We confirmed the data for demographic and self-reported comorbidity with participants at the 15-month follow-up visit.

All participants were interviewed by a group of certified doctors by telephone and were asked to complete a series of questionnaires, including a self-reported symptom questionnaire (shown in Appendix), the modified British Medical Research Council (mMRC) dyspnoea scale, psychological status questionnaire and Ischemic Stroke and Cardiovascular Events Registry. In the self-reported symptom questionnaire, participants were asked to report new and persistent symptoms or any more severe symptoms than before the onset of COVID-19(9). The mMRC dyspnea scale is a five-level scoring scale used to describe the degree of dyspnea caused by physical activity. A higher mMRC dyspnea scale score indicates more severe dyspnea (10).

Psychological conditions were measured using various scales: the GAD-7 anxiety scale (0–4 points for no anxiety disorder, 5–9 points for mild anxiety, 10–14 points for moderate anxiety, and 15–21 points for severe anxiety) (11), the PHQ-9 depression scale (0–4 points for no depression, 5–9 points for mild depression, 10–14 points for moderate depression, 15–19 points for moderate-severe depression, and 20–27 points for severe depression) (12) and the ASI scale for insomnia (0–7 points indicate no insomnia, 8–14 points indicate mild insomnia, 15–21 points indicate moderate insomnia and 22–28 points indicate severe insomnia) (13). We used the PC-PTSD (primary care PTSD screen) to identify PTSD symptoms, which was developed to quickly detect PTSD based on DSM-IV PTSD diagnostic criteria (14). The PC-PTSD included four items, and each item was designed to report whether the following symptoms were present or not, including reexperiencing, avoidance, hyperarousal and numbing. Answering “yes” was scored as 1, answering “no” as 0, and the scores of four items were added to get a total score. Generally, a total score of 3 or above is considered a positive result, indicating a clinically significant PTSD.

The EuroQol five-dimension five-level (EQ-5D-5L) questionnaire was used to assess patient quality of life by evaluating the following five factors: mobility, self-care, daily activities, pain or discomfort, and anxiety or depression (15). The classification of each element is divided into five levels, ranging from no problem to extreme problems. The post-COVID-19 functional status (PCFS) scale is recommended for use during the current COVID-19 pandemic (9). It is proposed that it could be used to display the direct retrieval and functional sequelae of COVID-19.

The follow-up was conducted from April 30 to May 9, 2021. A group of certified medical staff completed the follow-ups through telephone interviews. REDCap electronic data collection tools were used to manage the data, which helped to minimize missing inputs and allow for real-time data verification and quality control.

### Definition

Onset was defined as the date on which patients with confirmed COVID-19 first developed symptoms, excluding those with asymptomatic infection.

Severe cases are defined in accordance with the seventh edition of Chinese COVID-19 diagnosis and treatment guideline (16), which means that adults meet any of the following: Shortness of breath, RR > 30 times/min; In resting state, oxygen saturation when inhaling air degree of ≤ 93%; arterial partial pressure of oxygen (PaO2)/inhaled oxygen concentration (FiO2) ≤ 300 mmHg; progressive worsening of clinical symptoms, and lung imaging showed that the lesions progressed significantly within 24–48 h > 50%.

The new-onset diabetes mellitus in our study was based on glycated hemoglobin A1C (HbA1C) with a threshold of ≥6.5% or fasting plasma glucose of above 7.0 mmol/L. Deep venous thrombosis (DVT) was defined as forming a blood clot within a deep vein. The diagnosis of DVT of the lower limbs in our study was performed by duplex ultrasound imaging. Autoimmune thyroid disease (AITD) was defined as having thyroid antibodies that can be detected in the blood, including thyroglobulin antibodies, thyroid microsomal antibodies, and TSH receptor antibodies.

### Patient Outcomes

Primary outcomes included persistent symptoms (fatigue, sleep disturbance, cough, dyspnea, loss of taste, loss of smell, loss of appetite, hair loss, or photophobia) and psychological consequences (anxiety, depression, insomnia and PTSD as assessed by a series of standard scales) at the 15-month follow-up.

Secondary outcomes included health-related quality of life (pain or discomfort, anxiety or depression, mobility, personal nursing, and daily activities), PCFS scales, and all-cause death and extrapulmonary organ function (including major adverse cardiovascular events, deep vein thrombosis of the lower extremities, new-onset autoimmune thyroid disease, new-onset diabetes, and newly diagnosed cancer) at the 15-month follow-up.

### Statistical Analysis

Continuous variables are expressed as the median, and categorical variables are expressed as a percentage of the sum of absolute values. The participants were divided into two groups according to their symptom severity during hospitalization: severe and non-severe. We compared the demographic characteristics and long-term health outcomes of the two groups of participants. We also compared the long-term health outcomes of males and females. To compare the symptoms, physical activity, and health-related quality of life between men and women, we used the Mann–Whitney *U* test, χ^2^ test, or Fisher's exact test where appropriate.

The multivariate-adjusted logistic regression model was used to estimate the odds ratio (OR) and 95% confidence interval between disease severity and subtype outcome. For the relationship between disease severity and continuous outcome, a multivariate-adjusted linear regression model was used to estimate β estimates and 95% Cis. Confounding factors include age, sex, smoking (never smoker, current smoker, and former smoker), comorbidities (hypertension, diabetes, cardiovascular disease, cerebrovascular disease, malignant tumor, chronic obstructive pulmonary disease, and chronic kidney disease), corticosteroids, antiviral drugs (arbidol, chloroquine phosphate, and hydroxychloroquine), convalescent plasma therapy, and intravenous immunoglobulin.

Multivariate adjusted logistic regression analysis was used to explore the risk factors related to PTSD, ASI-sleep disorders, GAD-anxiety, and fatigue syndrome. Adverse mental outcomes occurred in ~20% of enrolled subjects, we followed accepted statistical practice and considered 10 variables in our multiple regression model. Variables associated with outcome measures (age, sex, comorbidities, severity of illness, corticosteroids, special oxygen therapy, length of hospital stay, symptoms remaining after discharge, and COVID-19 recovery status scale) were all included in the model. For the association of comorbidity with outcome, the above-mentioned variables except for disease severity were all included.

All the tests were two-sided, and a *P*-value of < 0.05 was considered statistically significant. We included all the follow-up participants in the final analysis without entering any missing data. All statistical analyses were performed using SAS version 9.4.

## Results

### Baseline Characteristics of the Study Population

The demographic and clinical characteristics of the participants are shown in Table 1. The median age of the enrolled patients was 62.0 (52.0–70.0) years old, with 239 males (44.8%) and 295 females (55.2%). The most common comorbidities were hypertension (198 patients, 37.08%), followed by diabetes (85 patients, 15.92%) and atherosclerotic cardio-cerebrovascular disease (ASCVD) (71 patients, 13.30%). A total of 403 (75.47%) of 534 participants required supplemental oxygen therapy during hospitalization, 15 (2.81%) required high-flow nasal oxygen inhalation (HFNC), non-invasive mechanical ventilation (non-IMV), or both, and 3 (0.56%) required extracorporeal membrane oxygenation (ECMO), IMV, or both. The median duration of hospitalization was 29.0 (17.0–40.0) days. The median time from symptom onset to follow-up was 460.0 (451.0–467.0) days, and the median time from discharge to follow-up was 414.0 (408.0–420.0) days (Table 1).

**Table 1:** Characteristics of 534 enrolled patients with COVID-19.

	**Total (*n* = 534)**	**Non-severe (*n* = 420)**	**Severe (*n* = 114)**	***P*-value**
Age, years				
Median (IQR)	62.0 (52.0, 70.0)	60.0 (50.0, 68.0)	70.0 (61.0, 78.0)	<0.0001
Distribution-*n* (%)				<0.0001
14–49	106 (19.85)	99 (23.57)	7 (6.14)	
50–64	205 (38.39)	177 (42.14)	28 (24.56)	
>65	223 (41.76)	144 (34.29)	79 (69.30)	
Sex				<0.0001
Male	239 (44.76)	169 (40.24)	70 (61.40)	
Female	295 (55.24)	251 (59.76)	44 (38.60)	
Cigarette smoking				0.0463
Never-smoker	293 (83.24)	236 (84.29)	57 (79.17)	
Current smoker	23 (6.53)	22 (7.86)	1 (1.39)	
Former smoker	36 (10.23)	22 (7.86)	14 (19.44)	
Comorbidities				
Hypertension	198 (37.08)	145 (34.52)	53 (46.49)	0.0190
Diabetes	85 (15.92)	61 (14.52)	24 (21.05)	0.0911
ASCVD	71 (13.30)	38 (9.05)	33 (28.95)	<0.0001
Asthma	2 (0.37)	2 (0.48)	0 (0.00)	1.0000
COPD	22 (4.12)	10 (2.38)	12 (10.53)	0.0003
Chronic kidney disease	7 (1.31)	2 (0.48)	5 (4.39)	0.0053
Chronic liver disease	24 (4.49)	19 (4.52)	5 (4.39)	0.9498
Cancer	23 (4.31)	16 (3.81)	7 (6.14)	0.4082
Highest seven-category scale during hospital stay				
3: not requiring supplemental oxygen	113 (21.16)	108 (25.71)	5 (4.39)	<0.0001
4: requiring supplemental oxygen	403 (75.47)	312 (74.29)	91 (79.82)	0.2229
5: requiring HFNC or non-IMV, or both	15 (2.81)	0 (0.00)	15 (13.16)	<0.0001
6: requiring ECMO or IMV, or both	3 (0.56)	0 (0.00)	3 (2.63)	0.0095
Admission into ICU	3		3	
Length of ICU hospitalization	32.0 (20.0–42.0)		32.0 (20.0–42.0)	
Treatment received during hospital stay				
Antivirals	499 (93.45)	394 (93.81)	105 (92.11)	0.5144
Antibiotics	231 (43.26)	160 (38.10)	71 (62.28)	<0.0001
Corticosteroids	35 (6.55)	16 (3.81)	19 (16.67)	<0.0001
Tocilizumab	11 (2.06)	0 (0.00)	11 (9.65)	<0.0001
Convalescent plasma therapy	20 (3.75)	11 (2.62)	9 (7.89)	0.0186
Intravenous immunoglobulin	18 (3.37)	9 (2.14)	9 (7.89)	0.0064
CRRT	1 (0.22)	0 (0.00)	1 (1.09)	0.1991
Length of hospital stay, days	29.0 (17.0, 40.0)	28.0 (17.0, 40.0)	30.50 (20.0, 42.0)	0.0354
Time from symptom onset to admission, days	12.0 (4.0, 26.0)	13.0 (4.0, 27.0)	8.0 (2.0, 22.0)	0.0033
Time form discharge to follow-up, days	414.0 (408.0, 420.0)	415.0 (409.0, 420.0)	411.0 (404.0, 419.0)	0.0016
Time form symptom onset to follow-up, days	460.0 (451.0, 467.0)	461.0 (451.0, 468.0)	456.0 (444.0, 467.0)	0.0123

*Data are n (%) or median (IQR). IQR, interquartile range; ASCVD, atherosclerotic cardio-cerebrovascular disease; COPD, chronic obstructive pulmonary disease; HFNC, high-flow nasal cannula for oxygen therapy; ECMO, extracorporeal membrane oxygenation; IMV, invasive mechanical ventilation; CRRT, continuous renal replacement therapy; COVID-19, corona virus disease 2019*.

### Persistent Symptoms and Psychological Consequences at the 15-Month Follow-Up

There were still many patients who had persistent symptoms. As shown in Table 2 and Supplementary Table S2, 44.57% of participants (238 of 534 patients) reported at least one symptom at follow-up, and a higher percentage was observed in women. The most common self-reported symptoms at 15 months after SARS-CoV-2 infection were sleep difficulties (99/534, 18.54%, Table 2) and fatigue (92/534, 17.23%), followed by memory loss (86/534, 16.10%). In addition, at 15 months after SARS-CoV-2 infection, 11.42% (61/534, Table 2) of patients still reported chest tightness, 9.93% (53/534) reported cough, and 8.05% (43/534) reported hair loss. A total of 5.43% (29/534) of patients reported dyspnea, 3.18% (17/534) reported smell disorder, 2.81% (15/534) reported taste disorder, and 2.62% (14/534) reported photophobia.

**Table 2:** Persistent symptoms and psychological consequences at 15-month follow-up.

	**Total (*n* = 534)**	**Non-severe (*n* = 420)**	**Severe (*n* = 114)**	***P***	OR or β (95%CI)^*^	***P* for regression**
Self-report symptoms—*n* (%)						
Any	238 (44.57)	175 (41.67)	63 (55.26)	0.0096	1.46 (0.92, 2.34)	0.1097
Sleep disorder	99 (18.54)	80 (19.05)	19 (16.67)	0.5618	0.81 (0.44, 1.48)	0.4902
Fatigue	92 (17.23)	68 (16.19)	24 (21.05)	0.2228	1.25 (0.69, 2.29)	0.4601
Memory loss	86 (16.10)	61 (14.52)	25 (21.93)	0.0564	1.34 (0.74, 2.43)	0.3399
Arthralgia	66 (12.36)	50 (11.90)	16 (14.04)	0.5399	1.30 (0.67, 2.49)	0.4354
Chest tightness	61 (11.42)	37 (8.81)	24 (21.05)	0.0003	2.55 (1.34, 4.87)	0.0046
Dizziness	55 (10.30)	37 (8.81)	18 (15.79)	0.0297	1.73 (0.87, 3.42)	0.1177
Cough	53 (9.93)	40 (9.52)	13 (11.40)	0.5517	0.93 (0.45, 1.95)	0.8574
Sore throat	52 (9.74)	35 (8.33)	17 (14.91)	0.0356	1.46 (0.71, 3.02)	0.3029
Headache	47 (8.80)	35 (8.33)	12 (10.53)	0.4636	1.37 (0.64, 2.93)	0.4251
Hair loss	43 (8.05)	34 (8.10)	9 (7.89)	0.9444	1.40 (0.59, 3.34)	0.4440
Myalgia	41 (7.68)	26 (6.19)	15 (13.16)	0.0132	2.14 (1.00, 4.60)	0.0506
Palpitation	37 (6.93)	24 (5.71)	13 (11.40)	0.0339	1.99 (0.88, 4.50)	0.0974
Chest pain	36 (6.74)	23 (5.48)	13 (11.40)	0.0252	2.63 (1.18, 5.86)	0.0180
Anorexia	31 (5.81)	20 (4.76)	11 (9.65)	0.0478	1.89 (0.79, 4.48)	0.1503
Dyspnea	29 (5.43)	18 (4.29)	11 (9.65)	0.0250	2.21 (0.92, 5.31)	0.0777
Diarrhea	25 (4.68)	23 (5.48)	2 (1.75)	0.0953	0.35 (0.08, 1.57)	0.1682
Rash	22 (4.12)	15 (3.57)	7 (6.14)	0.3379	1.87 (0.66, 5.29)	0.2410
Smell disorder	17 (3.18)	13 (3.10)	4 (3.51)	1.0000	1.54 (0.45, 5.31)	0.4923
Taste disorder	15 (2.81)	12 (2.86)	3 (2.63)	1.0000	0.72 (0.17, 2.97)	0.6480
photophobia	14 (2.62)	6 (1.43)	8 (7.02)	0.0029	6.93 (2.08, 23.11)	0.0016
Nausea or vomiting	10 (1.87)	7 (1.67)	3 (2.63)	0.7760	1.43 (0.31, 6.57)	0.6439
Intermittent fever	3 (0.56)	3 (0.71)	0 (0.00)	1.0000	0.00 (0.00, 454E94)	0.9361
mMRC				0.0124	0.45 (0.19, 1.07)	0.0702
mMRC4	3 (0.56)	0 (0.00)	3 (2.63)			
mMRC3	4 (0.75)	3 (0.71)	1 (0.88)			
mMRC2	6 (1.12)	4 (0.95)	2 (1.75)			
mMRC1	16 (3.00)	11 (2.62)	5 (4.39)			
mMRC0	505 (94.57)	402 (95.71)	103 (90.35)			
PHQ-9 scale of depression				0.0030	0.57 (0.30, 1.06)	0.0768
No depression	470 (88.01)	375 (89.29)	95 (83.33)			
Mild depression	37 (6.93)	30 (7.14)	7 (6.14)			
Moderate depression	12 (2.25)	9 (2.14)	3 (2.63)			
Severe depression	15 (2.81)	6 (1.43)	9 (7.89)			
GAD-7 scale of anxiety				0.0944	0.65 (0.31, 1.34)	0.2397
No anxiety	485 (90.82)	386 (91.90)	99 (86.84)			
Mild anxiety	36 (6.74)	27 (6.43)	9 (7.89)			
Moderate anxiety	6 (1.12)	4 (0.95)	2 (1.75)			
Severe anxiety	7 (1.31)	3 (0.71)	4 (3.51)			
ASI scale of insomnia				0.2412	1.25 (0.62, 2.53)	0.5321
No insomnia	464 (86.89)	364 (86.67)	100 (87.72)			
Mild insomnia	52 (9.74)	43 (10.24)	9 (7.89)			
Moderate insomnia	15 (2.81)	12 (2.86)	3 (2.63)			
Severe insomnia	3 (0.56)	1 (0.24)	2 (1.75)			
PTSD screen				0.0671	2.00 (0.77, 5.17)	0.1546
Negative	509 (95.32)	404 (96.19)	105 (92.11)			
Positive	25 (4.68)	16 (3.81)	9 (7.89)			

*Data are n (%) or median (IQR)*.

* *OR or β (95%CI) obtained by logistic regression, rank logistic regression and linear regression, adjusted for age, comorbidities, length of hospital stay, corticosteroid, 5: admitted to hospital, requiring HFNC or non-IMV or both, 6: admitted to hospital, requiring ECMO or IMV or both*.

*mMRC, modified British medical research council; PHQ-9, patient health questionnaire version 9; GAD-7, generalized anxiety disorder version 7; ASI, Arabic scale of insomnia; PTSD, post-traumatic stress disorder*.

The long-term impact of COVID-19 on the psychological consequences of patients after discharge from the hospital should not be ignored. As measured by the PHQ-9 and GAD-7 scales, 6.4% (34/534, Table 2) of patients had varying degrees of depression, and 9.2% (49/534) had different degrees of anxiety. According to the ASI questionnaire test, 13.0% (70/534) had various degrees of insomnia. The results from the PTSD screening scale showed that 4.7% (25/534) of patients had PTSD at 15 months after acute infection. The incidence rates of these adverse psychological conditions were higher in women than in men (see Supplementary Tables S2, S3, *P* < 0.05).

### Health-Related Quality of Life, PCFS Scales and All-Cause Death and Extrapulmonary Organ Function at the 15-Month Follow-Up

The results from the EQ-5D-5 L questionnaire showed that 19.10% (102/534) of the patients had trouble with mobility, 13.11% (70/534) had personal care problems, 15.92% (85/534) reported difficulties with performing their usual activities, 19.10% (102/534) had pain or discomfort and 20.79% (111/534) had anxiety or depression. The severe COVID-19 patients reported more problems in each sub-item of the EQ-5D-5L questionnaire and had worse quality of life than non-severe patients (all *P* < 0.05, Table 3).

**Table 3:** Health-related quality of life, PCFS scales and extrapulmonary organ function at 15-month follow-up.

	**Total (*n* = 534)**	**Non-severe (*n* = 420)**	**Severe (*n* = 114)**	***p***	OR or β (95%CI)^*^	***P* for regression**
Events in one-year after discharge—*n* (%)						
Non-fatal myocardial infarction or non-fatal stroke	7 (1.31)	4 (0.95)	3 (2.63)	0.3505	2.09 (0.39, 11.09)	0.3887
Heart failure hospitalization	5 (0.94)	1 (0.24)	4 (3.51)	0.0076	7.52 (0.66, 85.11)	0.1031
Arterial revascularization therapy	11 (2.06)	8 (1.90)	3 (2.63)	0.9102	0.43 (0.05, 3.74)	0.4449
New-onset venous thrombotic disease	9 (1.69)	7 (1.67)	2 (1.75)	1.0000	0.59 (0.10, 3.51)	0.5613
Exacerbation of renal disease requires dialysis or kidney transplantation	1 (0.19)	1 (0.24)	0 (0.00)	1.0000	0.00 (0.00, 161E90)	0.9413
New-onset diabetes	19 (3.56)	12 (2.86)	7 (6.14)	0.1635	2.12 (0.76, 5.91)	0.1491
New-onset AITD	10 (1.87)	9 (2.14)	1 (0.88)	0.6209	0.53 (0.06, 4.54)	0.5617
New-onset neuropsychiatric disease	3 (0.56)	1 (0.24)	2 (1.75)	0.1167	4.12 (0.29, 58.35)	0.2951
New-onset cancer	4 (0.75)	3 (0.71)	1 (0.88)	1.0000	0.00 (0.00, 6E166)	0.9524
EQ-5D-5L questionnaire						
Mobility: problems with walking	102 (19.10)	56 (13.33)	46 (40.35)	<0.0001	2.63 (1.52, 4.57)	0.0006
Personal care: problems with washing or dishing	70 (13.11)	34 (8.10)	36 (31.58)	<0.0001	2.88 (1.55, 5.36)	0.0008
Usual activity: problems with usual activity	85 (15.92)	47 (11.19)	38 (33.33)	<0.0001	2.29 (1.28, 4.09)	0.0052
Pain or discomfort	102 (19.10)	56 (13.33)	46 (40.35)	<0.0001	3.85 (2.25, 6.57)	<0.0001
Anxiety or depression	111 (20.79)	69 (16.43)	42 (36.84)	<0.0001	2.38 (1.41, 4.04)	0.0013
Quality of life	85.50 (80.00, 90.00)	89.00 (80.00, 90.50)	80.00 (70.00, 90.00)	0.0023	−4.99 (−9.02, −0.96)	0.0154
PCFS scale				0.0043	0.61 (0.39, 0.97)	0.0348
F0	348 (65.17)	291 (69.29)	57 (50.00)			
F1	13 (2.43)	10 (2.38)	3 (2.63)			
F2	13 (2.43)	9 (2.14)	4 (3.51)			
F3	95 (17.79)	66 (15.71)	29 (25.44)			
F4	65 (12.17)	44 (10.48)	21 (18.42)			

*Data are n (%)*.

* *OR or β (95%CI) obtained by logistic regression, rank logistic regression and linear regression, adjusted for age, comorbidities, length of hospital stay, corticosteroid, 5: admitted to hospital, requiring HFNC or non-IMV or both, 6: admitted to hospital, requiring ECMO or IMV or both*.

*AITD, autoimmune thyroid disease; EQ-5D-5L, EuroQol 5-Dimension Questionnaire 5-level version; PCSF, post-COVID-19 functional status*.

The PCSF rating results showed that 65.17% (348/534) of patients recovered well in functional status, reaching the F0 grade. That means 65.17% of the patients were able to recover to their pre-sick condition, and their life and work were not affected by COVID-19. There was no significant difference in the proportion of F0 grade individuals between severe patients and non-severe patients (*P* > 0.05, Table 3).

Notably, 15 patients died after discharge. The primary reason was the deterioration of lung, heart, and kidney conditions. The detailed characteristics are shown in Supplementary Table S1. In addition, seven patients reported non-fatal myocardial infarctions or ischemic strokes after discharge. Five patients were readmitted for hospitalization again due to heart failure. Eleven patients underwent arterial revascularization or stent implantation. Nine patients suffered from acute pulmonary embolism due to deep lower limb venous thrombosis. One patient underwent dialysis treatment due to worsening renal failure. Nineteen patients were diagnosed with new-onset diabetes, ten reported new-onset autoimmune thyroid disease, and four were newly diagnosed with malignant tumors.

### Risk Factors for Long-Term Health Impacts and Their Association With Disease Severity

After adjusting for confounding factors such as age, sex, smoking, comorbidities, length of stay, oxygen therapy, and medication, the risk of chest tightness, chest pain, and photophobia in severe patients was still significantly higher than that of non-severe patients, with OR values of 2.55 (95% CI 1.34–4.87, Table 2), 2.63 (1.18–5.86) and 6.93 (2.08–23.11), respectively. However, the risk of fatigue and sleep disturbance in severe patients was not significant, and the OR values were 1.25 (95% CI 0.69–2.29, Table 2) and 0.81 (0.44–1.48), respectively. There was no significant difference in the proportion of cough, dyspnea, hair loss, smell disorder, or taste disorder between severe and non-severe patients (*P* > 0.05, Table 2).

**Figure 2:**
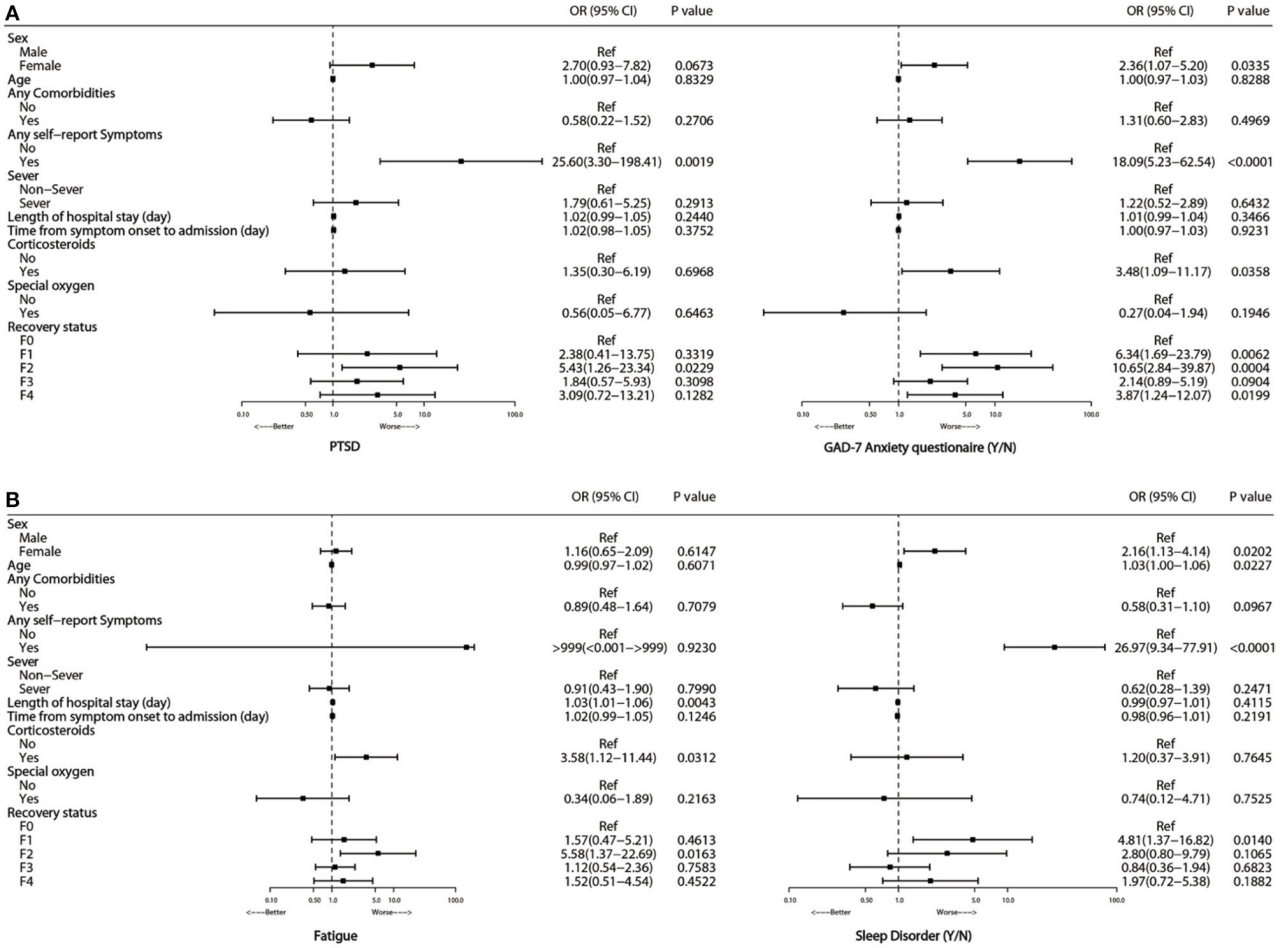
Risk factors associated with PTSD and GAD-7 anxiety **(A)**, fatigue and sleep disorder **(B)** at 15-month follow-up.

Multivariate adjusted logistic regression analysis showed that glucocorticoid treatment during hospitalization (OR 3.58, 95%CI 1.12–11.44, *P* = 0.0312, Figure 2) was significantly associated with an increased risk of fatigue and GAD-7 anxiety score (OR 3.48, 95%CI 1.09–11.17, *P* = 0.0358, Figure 2). No significant associations were observed between fatigue syndromes and age, gender or disease severity.

Multivariate adjusted logistic regression analysis showed that gender and the presence of self-reported symptoms were significantly associated with adverse mental consequences. Compared with men, women had an OR of 2.7 (95% CI 0.93–7.28, Figure 2) for PTSD, an OR of 2.36 (1.07–5.20) for GAD-7 anxiety, and an OR of 2.16 (1.13–4.14) for ASI sleep disorder. Participants with self-reported symptoms showed OR values of 25.6 (95% CI 3.3–198.4) for PTSD, 18.09 (5.23–62.54) for GAD-7 anxiety, and 26.97 (9.34–77.91) for ASI sleep disorder compared with participants without self-reported symptoms. No apparent associations were observed between age or disease severity and PTSD, GAD-7 anxiety, or ASI-sleep disorder.

## Discussion

In this study, we reported the health outcomes of patients who were hospitalized with COVID-19 at 15 months after acute infection during the first pandemic in Wuhan, China. To our knowledge, this is the longest follow-up cohort study of hospitalized COVID-19 patients.

Our data showed that the most common persistent symptoms at 15 months after onset were sleep difficulties and fatigue, followed by memory loss, chest tightness and cough. Compared with the published data of this cohort 3 months after discharge (8), most of the acute symptoms of COVID-19 patients were significantly relieved or even disappeared, and no serious respiratory complications remained. This is consistent with the data of the previous 12-month long-term follow-up study of COVID-19 (7). Our data also showed that residual psychological problems remain prominent. At the 15-month follow-up, 6.4% (34/534) of the patients had depression, 9.2% (49/534) had anxiety, 13.0% (70/534) had insomnia and 4.7% (25/534) had PTSD. These results suggested that the psychological consequences of long-term COVID-19 should be noted.

Compared to the general public, patients that were infected by COVID-19 have in fact a higher risk of developing these adverse mental and psychological symptoms. The prevalence of generalized anxiety disorder (GAD) in adult was very common both in community and in clinic. According to the review of epidemiological studies in Europe, the 12-month prevalence rate of GAD was 1.7–3.4% (17) and the lifetime prevalence was 4.3–5.9% (18). The prevalence rate of GAD in COVID-19 patients in this study was 9.2%, which was significantly higher than that of the general public. Data from a multi-country study involving 252,503 cases from 68 countries showed that the 1-year prevalence rate of mild depression was 2.8% (19). Another community survey in Taiwan, China, including 5,664 individuals aged ≥55, showed that the prevalence rate of mild depression was 4% (20, 21). In our study, the prevalence rate of depression in COVID-19 patients reached 6.4%, which was also significantly higher than that of the general public. In the sample from general adult population in the United States and Canada, the 1-year prevalence rate of PTSD is 3.5–4.7 (22, 23). The prevalence rate of PTSD in COVID-19 patients in this cohort was as high as 25% at 3 months after discharge (data not published). Although the prevalence rate of PTSD at 15 months after onset has dropped to 4.7%, it is still in the high level when compared with that in the general public. Given all of that, COVID-19 patients still have a higher risk of adverse mental and psychological illness, even 15 months after onset.

Unlike individual-level traumatic events, the COVID-19 outbreak has been a continuing crisis for every member of society (24). Globally, the epidemic has led to an increase of about 53 million in the incidence of depression in 2020, an increase of about 27.6% (25).

Furthermore, we attempted to analyze the potential risk factors related to health outcomes and the relationship with the severity of the disease. Our data demonstrated that female COVID-19 patients were more likely to have residual symptoms, such as fatigue and sleep disorders, and a range of adverse psychological and psychiatric consequences. Patients with long-term legacy symptoms are more likely to develop PTSD. Before the outbreak, women had higher rates of depression and anxiety disorders than men worldwide (25). In China, the prevalence of any depressive disorder in women is higher than that in men, and its lifetime prevalence is 1.44 times that of men (26). After the outbreak of pandemic, an even greater difference in mental disorder prevalence was found between the two genders, which was speculated that females are more likely to be affected by the social and economic consequences of the pandemic (27–29).

In addition, our study first showed that the use of glucocorticoids during hospitalization was significantly related to an increased risk of chronic fatigue and anxiety in patients with COVID-19 after discharge. High-dose corticosteroids were administered to many critically ill patients in Wuhan (30) and were associated with higher mortality risk. Previous research on SARS patients found that high-dose corticosteroid use could lead to osteonecrosis of the femoral head (OFNH) (31). Unfortunately, we were unable to obtain specific dose and use time of each patient in this cohort which limits the conclusions we can draw from these data. Future studies are urgently needed that are specially designed to address the relation between glucocorticoid use and adverse psychological outcomes.

The underlying mechanism of long COVID-19 is complicated and cannot be simply attributed to SARS-CoV-2 infection. The pathogenesis of psychiatric symptoms and disorders that arise during the COVID-19 pandemic may include biologic and psychosocial factors.

On one hand, several retrospective studies also suggest that COVID-19 may affect the brain (32, 33). In addition, a literature review demonstrated that past viral epidemics were associated with neuropsychiatric symptoms such as demyelination, encephalopathy, and neuromuscular dysfunction, as well as mood changes and psychosis (34). The symptoms occurred during infection or following recovery from the infection in the ensuing weeks, months, or longer. Multiple studies suggest that COVID-19 may indirectly affect the central nervous system through the associated inflammatory immune response and medical interventions that are administered (32, 33, 35). Immunologic findings in patients with COVID-19 include elevated serum C-reactive protein and pro-inflammatory cytokines (e.g., IL-6) and decreased total blood lymphocyte counts (34). Critical illness and resultant intensive care unit stays commonly expose patients to extreme physiological and psychological stressors that are life-threatening and traumatic, and frequently precipitate persistent psychiatric illness (35, 36).

On the other hand, psychiatric illnesses that occur during the pandemic may stem from psychosocial factors such as (37–41): frequency and extent of exposure to individuals infected with the virus, fear of infecting family members, fear of being discriminated against, lack of access to testing and medical care for COVID-19, physical distancing, home confinement, quarantining, and loneliness, shortages of available resources (e.g., personal protective equipment), diminished personal freedoms, continuous media reporting about the pandemic and the uncertainty surrounding its eventual outcome. The role of those mentioned above social and psychological factors is particularly serious in Wuhan, where the first outbreak occurred. Thus, psychological and social intervention of this disease carries great importance for the COVID-19 patients in recovery phase. The rehabilitation of COVID-19 patients is a long-term and systematic project. Our research will help inform decision-making on care service design and priorities for these patients.

We also investigated the long-term performance of extrapulmonary organs and deaths during follow-up. For example, it has been observed that some patients have new-onset diabetes, are newly diagnosed with AITD, and have venous thromboembolic diseases, including cardiovascular and cerebrovascular events. The receptor angiotensin-converting enzyme 2 (ACE2), which modulates the invasion of SARS-CoV-2 into the body, is also expressed in many vital metabolic organs and tissues, including pancreatic β cells (42), adipose tissue (43), intestines (44), and kidneys (44); SARS-CoV-2 infection may cause pleiotropic changes in glucose metabolism, complicate the pathophysiology of existing diabetes, or cause new hyperglycemia or new diabetes (45). There have been some precedents of ketosis-prone diabetes caused by coronaviruses. A previous study showed that the incidence of high fasting blood glucose and acute new-onset diabetes in SARS coronavirus pneumonia patients is higher than that in non-SARS patients (46). Our study showed that 3.5% (19/534) of the patients had a new fasting blood glucose of >7 mmol/L or HbA1c ≥ 6.5% at the 15-month follow-up and had no previous history of diabetes. We deduced that COVID-19 has potential diabetic effects.

This study has several limitations. First, for the new symptoms that appeared after COVID-19, there was no further stratification to determine whether the symptoms continued after COVID-19, worsened after COVID-19 recovery, or occurred after discharge from the hospital. Second, the cases included in this study were all hospitalized COVID-19 patients, with a lack of data from outpatients. Lastly, this is a single-center study in a specific region which challenges the generalizability of the study findings. We are in urgent need of multi-center studies covering a wider range of patient cohorts over different regions especially when describing the causes of a pandemic affecting the entire world population.

In conclusion, we conducted a 15-month follow-up and reported the persistent symptoms and psychological conditions in a COVID-19 patient cohort in Wuhan. Relevant risk factors, such as female gender and use of glucocorticoids for long COVID, were identified. All these findings were of great significance for managing COVID-19 patients during the long-term rehabilitation period.

## Data Availability Statement

The original contributions presented in the study are included in the article/Supplementary Material, further inquiries can be directed to the corresponding author/s. The data in this study can be shared with qualified researchers who submit a proposal with a valuable research question.

## Author Contributions

L-LS, Y-HB, HH, and W-FX designed the study and revised the manuscript. L-LS, JW, and Y-SW drafted the manuscript. L-LS, Y-SW, and XP performed the analysis. JL, HL, Y-RJ, XZ, LL, G-CL, J-WZ, WW, Y-JW, Z-HW, H-BS, S-SC, J-LC, and Z-WX collected the data. L-LS, Y-SW, and Y-HB designed the electronic follow-up questionnaire form. All authors had full access to all the data in the study, and they took responsibility for the integrity of the data and the accuracy of the data analysis.

## Conflict of Interest

The authors declare that the research was conducted in the absence of any commercial or financial relationships that could be construed as a potential conflict of interest.

## Publisher's Note

All claims expressed in this article are solely those of the authors and do not necessarily represent those of their affiliated organizations, or those of the publisher, the editors and the reviewers. Any product that may be evaluated in this article, or claim that may be made by its manufacturer, is not guaranteed or endorsed by the publisher.

## References

[1] Zhu N Zhang D Wang W Li X Yang B Song J. A novel coronavirus from patients with pneumonia in China, 2019. N Engl J Med. (2020) 382:727–33. 10.1056/NEJMoa2001017 31978945 PMC7092803

[2] World Health Organization. WHO Coronavirus (COVID-19) Dashboard. (2022). https://covid19.who.int/ (accessed March 12, 2022).

[3] Gupta A Madhavan MV Sehgal K Nair N Mahajan S Sehrawat TS. Extrapulmonary manifestations of COVID-19. Nat Med. (2020) 26:1017–32. 10.1038/s41591-020-0968-3 32651579 PMC11972613

[4] Guan WJ Ni ZY Hu Y Liang WH Ou CQ He JX. Clinical characteristics of coronavirus disease 2019 in China. N Engl J Med. (2020) 382:1708–20. 10.1056/NEJMoa2002032 32109013 PMC7092819

[5] Mandal S Barnett J Brill SE Brown JS Denneny EK Hare SS. ‘Long-COVID': a cross-sectional study of persisting symptoms, biomarker and imaging abnormalities following hospitalisation for COVID-19. Thorax. (2021) 76:396–8. 10.1136/thoraxjnl-2020-215818 33172844 PMC7615158

[6] Huang C Huang L Wang Y Li X Ren L Gu X. 6-month consequences of COVID-19 in patients discharged from hospital: a cohort study. Lancet. (2021) 397:220–32. 10.1016/S0140-6736(20)32656-8 33428867 PMC7833295

[7] Huang L Yao Q Gu X Wang Q Ren L Wang Y. 1-year outcomes in hospital survivors with COVID-19: a longitudinal cohort study. Lancet. (2021) 398:747–58. 10.1016/S0140-6736(21)01755-4 34454673 PMC8389999

[8] Sun LL Wang J Wang YS Hu PF Zhao ZQ Chen W. Symptomatic features and prognosis of 932 hospitalized patients with coronavirus disease 2019 in Wuhan. J Dig Dis. (2021) 22:271–81. 10.1111/1751-2980.12983 33742780 PMC8251293

[9] Klok FA Boon G Barco S Endres M Geelhoed JJM Knauss S. The Post-COVID-19 Functional Status scale: a tool to measure functional status over time after COVID-19. Eur Respir J. (2020) 56:2001494. 10.1183/13993003.01494-2020 32398306 PMC7236834

[10] Doherty DE Belfer MH Brunton SA Fromer L Morris CM Snader TC. Chronic obstructive pulmonary disease: Consensus recommendations for early diagnosis and treatment. J Fam Pract. (2006) 55:S1–8.

[11] Plummer F Manea L Trepel D McMillan D. Screening for anxiety disorders with the GAD-7 and GAD-2: a systematic review and diagnostic metaanalysis. Gen Hosp Psychiatry. (2016) 39:24–31. 10.1016/j.genhosppsych.2015.11.005 26719105

[12] Yu X Tam WW Wong PT Lam TH Stewart SM. The Patient Health Questionnaire-9 for measuring depressive symptoms among the general population in Hong Kong. Compr Psychiatry. (2012) 53:95–102. 10.1016/j.comppsych.2010.11.002 21193179

[13] Soldatos CR Dikeos DG Paparrigopoulos TJ. Athens Insomnia Scale: validation of an instrument based on ICD-10 criteria. J Psychosom Res. (2000) 48:555–60. 10.1016/S0022-3999(00)00095-7 11033374

[14] Prins A Ouimette P Kimerling R Camerond R Hugelshofer D Shaw-Hegwer J. The primary care PTSD screen (PC-PTSD): development and operating characteristics. Int J Psychiatry Clin Pract. (2004) 9:9–14. 10.1185/13552570312500236031158781

[15] Herdman M Gudex C Lloyd A Janssen M Kind P Parkin D. Development and preliminary testing of the new five-level version of EQ-5D (EQ-5D-5L). Qual Life Res. (2011) 20:1727–36. 10.1007/s11136-011-9903-x 21479777 PMC3220807

[16] Wei P-F. Diagnosis and treatment protocol for novel coronavirus pneumonia (Trial version 7). Chin Med J (Engl). (2020) 133:1087–95. 10.1097/CM9.0000000000000819 32358325 PMC7213636

[17] Wittchen HU Jacobi F Rehm J Gustavsson A Svensson M Jönsson B. The size and burden of mental disorders and other disorders of the brain in Europe 2010. Eur Neuropsychopharmacol. (2011) 21:655–79. 10.1016/j.euroneuro.2011.07.018 21896369

[18] Wittchen HU Jacobi F. Size and burden of mental disorders in Europe–a critical review and appraisal of 27 studies. Eur Neuropsychopharmacol. (2005) 15:357–76. 10.1016/j.euroneuro.2005.04.012 15961293

[19] Ayuso-Mateos JL Nuevo R Verdes E Naidoo N Chatterji S. From depressive symptoms to depressive disorders: the relevance of thresholds. Br J Psychiatry. (2010) 196:365–71. 10.1192/bjp.bp.109.071191 20435961

[20] Karlsson B Johnell K Sigström R Sjöberg L Fratiglioni L. Depression and depression treatment in a population-based study of individuals over 60 years old without dementia. Am J Geriatr Psychiatry. (2016) 24:615–23. 10.1016/j.jagp.2016.03.009 27297634

[21] Wu CS Yu SH Lee CY Tseng HY Chiu YF Hsiung CA. Prevalence of and risk factors for minor and major depression among community-dwelling older adults in Taiwan. Int Psychogeriatr. (2017) 29:1113–21. 10.1017/S1041610217000199 28390440

[22] Goldstein RB Smith SM Chou SP Saha TD Jung J Zhang H. The epidemiology of DSM-5 posttraumatic stress disorder in the United States: results from the National Epidemiologic Survey on Alcohol and Related Conditions-III. Soc Psychiatry Psychiatr Epidemiol. (2016) 51:1137–48. 10.1007/s00127-016-1208-5 27106853 PMC4980174

[23] Kessler RC Chiu WT Demler O Merikangas KR Walters EE. Prevalence, severity, and comorbidity of 12-month DSM-IV disorders in the National Comorbidity Survey Replication. Arch Gen Psychiatry. (2005) 62:617–27. 10.1001/archpsyc.62.6.617 15939839 PMC2847357

[24] Tang W Hu T Hu B Jin C Wang G Xie C. Prevalence and correlates of PTSD and depressive symptoms one month after the outbreak of the COVID-19 epidemic in a sample of home-quarantined Chinese university students. J Affect Disord. (2020) 274:1–7. 10.1016/j.jad.2020.05.009 32405111 PMC7217769

[25] COVID-19 Mental Disorders Collaborators. Global prevalence and burden of depressive and anxiety disorders in 204 countries and territories in 2020 due to the COVID-19 pandemic. Lancet. (2021) 398:1700–12. 10.1016/S0140-6736(21)02143-7 34634250 PMC8500697

[26] Lu J Xu X Huang Y Li T Ma C Xu G. Prevalence of depressive disorders and treatment in China: a cross-sectional epidemiological study. Lancet Psychiatry. (2021) 8:981–90. 10.1016/S2215-0366(21)00251-0 34559991

[27] United Nations. Policy Brief: The Impact of COVID-19 on Women. (2020). Available online at: https://www.un.org/sexualviolenceinconflict/wp-content/uploads/2020/06/report/policy-brief-the-impact-of-covid-19-on-women/policy-brief-the-impact-of-covid-19-on-women-en-1.pdf (accessed March 24, 2022).

[28] Wenham C Smith J Davies SE Feng H Grépin KA Harman S. Women are most affected by pandemics - lessons from past outbreaks. Nature. (2020) 583:194–8. 10.1038/d41586-020-02006-z 32641809

[29] Burki T. The indirect impact of COVID-19 on women. Lancet Infect Dis. (2020) 20:904–5. 10.1016/S1473-3099(20)30568-5 32738239 PMC7836874

[30] Zhou F Yu T Du R Fan G Liu Y Liu Z. Clinical course and risk factors for mortality of adult inpatients with COVID-19 in Wuhan, China: a retrospective cohort study. Lancet. (2020) 395:1054–62. 10.1016/S0140-6736(20)30566-3 32171076 PMC7270627

[31] Zhao FC Guo KJ Li ZR. Osteonecrosis of the femoral head in SARS patients: seven years later. Eur J Orthop Surg Traumatol. (2013) 23:671–7. 10.1007/s00590-012-1054-4 23412187

[32] Helms J Kremer S Merdji H Clere-Jehl R Schenck M Kummerlen C. Neurologic features in severe SARS-CoV-2 infection. N Engl J Med. (2020) 382:2268–70. 10.1056/NEJMc2008597 32294339 PMC7179967

[33] Romero-Sánchez CM Díaz-Maroto I Fernández-Díaz E Sánchez-Larsen Á Layos-Romero A García-García J. Neurologic manifestations in hospitalized patients with COVID-19: the ALBACOVID registry. Neurology. (2020) 95:e1060–70. 10.1212/WNL.0000000000009937 32482845 PMC7668545

[34] Troyer EA Kohn JN Hong S. Are we facing a crashing wave of neuropsychiatric sequelae of COVID-19? Neuropsychiatric symptoms and potential immunologic mechanisms. Brain Behav Immun. (2020) 87:34–9. 10.1016/j.bbi.2020.04.027 32298803 PMC7152874

[35] Rogers JP Chesney E Oliver D Pollak TA McGuire P Fusar-Poli P. Psychiatric and neuropsychiatric presentations associated with severe coronavirus infections: a systematic review and meta-analysis with comparison to the COVID-19 pandemic. Lancet Psychiatry. (2020) 7:611–27. 10.1016/S2215-0366(20)30203-0 32437679 PMC7234781

[36] Roy-Byrne P Stein MB. PTSD and Medical Illness. Post-Trauma Str Dis. New York, NY: Oxford University Press (2018). 10.1093/med/9780190259440.003.0005

[37] Xiang YT Jin Y Cheung T. Joint international collaboration to combat mental health challenges during the coronavirus disease 2019 pandemic. JAMA Psychiatry. (2020) 77:989–90. 10.1001/jamapsychiatry.2020.1057 32275289

[38] Galea S Merchant RM Lurie N. The mental health consequences of COVID-19 and physical distancing: the need for prevention and early intervention. JAMA Intern Med. (2020) 180:817–8. 10.1001/jamainternmed.2020.1562 32275292

[39] Pfefferbaum B North CS. Mental health and the Covid-19 pandemic. N Engl J Med. (2020) 383:510–2. 10.1056/NEJMp2008017 32283003

[40] Reger MA Stanley IH Joiner TE. Suicide mortality and coronavirus disease 2019-a perfect storm? JAMA Psychiatry. (2020) 77:1093–4. 10.1001/jamapsychiatry.2020.1060 32275300

[41] Brooks SK Webster RK Smith LE Woodland L Wessely S Greenberg N. The psychological impact of quarantine and how to reduce it: rapid review of the evidence. Lancet. (2020) 395:912–20. 10.1016/S0140-6736(20)30460-8 32112714 PMC7158942

[42] Fignani D Licata G Brusco N Nigi L Grieco GE Marselli L. SARS-CoV-2 receptor angiotensin i-converting enzyme type 2 (ACE2) is expressed in human pancreatic β-cells and in the human pancreas microvasculature. Front Endocrinol. (2020) 11:596898. 10.3389/fendo.2020.596898 33281748 PMC7691425

[43] Gheblawi M Wang K Viveiros A Nguyen Q Zhong JC Turner AJ. Angiotensin-converting enzyme 2: SARS-CoV-2 receptor and regulator of the renin-angiotensin system: celebrating the 20th anniversary of the discovery of ACE2. Circ Res. (2020) 126:1456–74. 10.1161/CIRCRESAHA.120.317015 32264791 PMC7188049

[44] Hamming I Timens W Bulthuis ML Lely AT Navis G van Goor H. Tissue distribution of ACE2 protein, the functional receptor for SARS coronavirus. A first step in understanding SARS pathogenesis. J Pathol (2004) 203:631–7. 10.1002/path.1570 15141377 PMC7167720

[45] Rubino F Amiel SA Zimmet P Alberti G Bornstein S Eckel RH. New-onset diabetes in Covid-19. N Engl J Med. (2020) 383:789–90. 10.1056/NEJMc2018688 32530585 PMC7304415

[46] Kothandaraman N Rengaraj A Xue B Yew WS Velan SS Karnani N. COVID-19 endocrinopathy with hindsight from SARS. Am J Physiol Endocrinol Metab. (2021) 320:E139–50. 10.1152/ajpendo.00480.2020 33236920 PMC7816429

