# Supplementary material for: 15-Month Health Outcomes and the Related Risk Factors of Hospitalized COVID-19 Patients From Onset: A Cohort Study
Source: Front Med (Lausanne). 2022 May 11;9:854788. doi: 10.3389/fmed.2022.854788 (PMC9131089; doi:10.3389/fmed.2022.854788)
Supplement: Supplementary file 1 [file Data_Sheet_1.docx]

**Supplementary appendix**

**15-month health outcomes and the related risk factors of hospitalized COVID-19 patients from onset: a cohort study**

Liang-Liang Sun, Jian Wang, Yu-Sheng Wang, Xiao Pan, Yu-shi Hao, Jun-Luo, Hua Liu, Yi-Rou Jiang, Xin Zhuang, Liang Lin, Gan-Cheng Li, Jun-Wei Zhao, Wei Wang, Yuan-Jin Wang, Zhi-Hao Wang, Hong-Biao Shan, Shuai-Shuai Chen, Jun-Lin Chen, Zhao-Wei Xu, Yong-Hai Bai, Hai Huang, Wei-Fen Xie

**Table of contents**

[Table S1. Characteristics of patients who died after discharge from hospital 3](#_Toc98483787)

[Table S2. Persistent symptoms and psychological consequences at 15-month follow-up according to sex 4](#_Toc98483788)

[Table S3. Health-related quality of life, PCFS scales and extrapulmonary organ function at 15-month follow-up according to sex 7](#_Toc98483789)

[Table S4. Change of PTSD outcomes among COVID-19 patients between 3-month and 15-month follow-up 9](#_Toc98483790)

[Table S5. Characteristics of COVID-19 patients with and without complete follow-up. 10](#_Toc98483791)

[COVID-19 patients 15-month follow-up questionnaire 11](#_Toc98483792)

# Table S1. Characteristics of patients who died after discharge from hospital

| No. | Age, years | Sex | Comorbidity | Severity | Highest seven category scale during hospitalization | Cause of death |
| --- | --- | --- | --- | --- | --- | --- |
| 1 | 87 | Female | Hypertension, diabetes | Severe | 5 | Acute complications of diabetes |
| 2 | 74 | Male | Hypertension, Myocardial infarction | Severe | 6 | Acute myocardial infarction |
| 3 | 85 | Male | Hypertension | Severe | 6 | COVID-19 |
| 4 | 70 | Male | None | Severe | 4 | Stroke |
| 5 | 87 | Female | Diabetes | Severe | 5 | Natural death |
| 6 | 90 | Female | None | Severe | 4 | Natural death |
| 7 | 72 | Male | COPD | Severe | 4 | Respiratory failure |
| 8 | 79 | Male | Stroke | Severe | 4 | COVID-19 |
| 9 | 86 | Female | ASCVD, Hypertension, Diabetes | Severe | 5 | multiple organ dysfunction syndrome |
| 10 | 61 | Male | COPD, Hypertension | Severe | 4 | Background disease |
| 11 | 57 | Male | Hypertension, Diabetes, CKD-4 | Severe | 4 | Background disease |
| 12 | 83 | Female | Hypertension, ASCVD | Severe | 4 | COVID-19 |
| 13 | 72 | Male | Hypertension | Mild | 4 | Interstitial pneumonia |
| 14 | 62 | Female | Chronic myeloid leukemia | Mild | 3 | pneumonia |
| 15 | 73 | Male | None | Mild | 3 | Interstitial pneumonia |

COPD=chronic obstructive pulmonary disease. ASCVD=atherosclerotic cardiovascular disease. CKD=chronic kidney disease.

#

# Table S2. Persistent symptoms and psychological consequences at 15-month follow-up according to sex

|  | | Total  (n=534) | Male  (n=239) | Female  (n=295) | p | OR or β(95%CI)* | P for regression |
| --- | --- | --- | --- | --- | --- | --- | --- |
| Self-Symptoms - n/N(%) | |  |  |  |  |  |  |
| Any | | 238(44.57) | 98(41.00) | 140(47.46) | 0.1357 | 1.40(0.97,2.01) | 0.0695 |
| Fever | | 3(0.56) | 0(0.00) | 3(1.02) | 0.3265 | 29119(0.00,2E119) | 0.9393 |
| Cough | | 53(9.93) | 22(9.21) | 31(10.51) | 0.6164 | 1.10(0.61,2.00) | 0.7498 |
| Dyspnea | | 29(5.43) | 11(4.60) | 18(6.10) | 0.4472 | 1.53(0.65,3.59) | 0.3295 |
| Fatigue | | 92(17.23) | 35(14.64) | 57(19.32) | 0.1546 | 1.49(0.92,2.43) | 0.1062 |
| Memory loss | | 86(16.10) | 29(12.13) | 57(19.32) | 0.0246 | 1.86(1.12,3.08) | 0.0168 |
| Sleep difficulties | | 99(18.54) | 32(13.39) | 67(22.71) | 0.0058 | 1.72(1.07,2.78) | 0.0265 |
| Hair loss | | 43(8.05) | 12(5.02) | 31(10.51) | 0.0205 | 2.52(1.20,5.28) | 0.0145 |
| Sore throat | | 52(9.74) | 19(7.95) | 33(11.19) | 0.2097 | 1.57(0.84,2.94) | 0.1568 |
| Smell disorder | | 17(3.18) | 6(2.51) | 11(3.73) | 0.4252 | 1.50(0.52,4.33) | 0.4489 |
| Taste disorder | | 15(2.81) | 3(1.26) | 12(4.07) | 0.0505 | 2.92(0.75,11.37) | 0.1215 |
| Anorexia | | 31(5.81) | 10(4.18) | 21(7.12) | 0.1493 | 1.84(0.82,4.14) | 0.1396 |
| Nausea or vomiting | | 10(1.87) | 5(2.09) | 5(1.69) | 0.9875 | 0.75(0.21,2.70) | 0.6569 |
| Diarrhea | | 25(4.68) | 11(4.60) | 14(4.75) | 0.9379 | 0.84(0.37,1.93) | 0.6845 |
| Palpitation | | 37(6.93) | 11(4.60) | 26(8.81) | 0.0567 | 2.54(1.14,5.63) | 0.0221 |
| Chest tightness | | 61(11.42) | 20(8.37) | 41(13.90) | 0.0458 | 2.29(1.23,4.27) | 0.0090 |
| Chest pain | | 36(6.74) | 11(4.60) | 25(8.47) | 0.0760 | 2.23(1.01,4.92) | 0.0474 |
| Myalgia | | 41(7.68) | 14(5.86) | 27(9.15) | 0.1550 | 1.81(0.89,3.67) | 0.1014 |
| Arthralgia | | 66(12.36) | 29(12.13) | 37(12.54) | 0.8866 | 0.98(0.57,1.69) | 0.9534 |
| Headache | | 47(8.80) | 16(6.69) | 31(10.51) | 0.1219 | 1.68(0.87,3.23) | 0.1217 |
| Dizziness | | 55(10.30) | 19(7.95) | 36(12.20) | 0.1078 | 1.78(0.96,3.32) | 0.0680 |
| Rash | | 22(4.12) | 9(3.77) | 13(4.41) | 0.7109 | 1.33(0.54,3.26) | 0.5329 |
| photophobia | | 14(2.62) | 5(2.09) | 9(3.05) | 0.4905 | 2.39(0.70,8.09) | 0.1620 |
| mMRC | |  |  |  | 0.5313 | 0.66(0.28,1.54) | 0.3351 |
| mMRC 4 | | 3(0.56) | 1(0.42) | 2(0.68) |  |  |  |
| mMRC 3 | | 4(0.75) | 1(0.42) | 3(1.02) |  |  |  |
| mMRC 2 | | 6(1.12) | 4(1.67) | 2(0.68) |  |  |  |
| mMRC 1 | | 16(3.00) | 5(2.09) | 11(3.73) |  |  |  |
| mMRC 0 | | 505(94.57) | 228(95.40) | 277(93.90) |  |  |  |
| PHQ-9 scale of depression |  | |  |  | 0.0150 | 0.35(0.19,0.64) | 0.0007 |
| No depression | 470(88.01) | | 222(92.89) | 248(84.07) |  |  |  |
| Mild depression | 37(6.93) | | 10(4.18) | 27(9.15) |  |  |  |
| Moderate depression | 12(2.25) | | 2(0.84) | 10(3.39) |  |  |  |
| Severe depression | 15(2.81) | | 5(2.09) | 10(3.39) |  |  |  |
| GAD-7 scale of anxiety |  | |  |  | 0.0011 | 0.38(0.19,0.75) | 0.0058 |
| No anxiety | 485(90.82) | | 226(94.56) | 259(87.80) |  |  |  |
| Mild anxiety | 36(6.74) | | 5(2.09) | 31(10.51) |  |  |  |
| Moderate anxiety | 6(1.12) | | 4(1.67) | 2(0.68) |  |  |  |
| Severe anxiety | 7(1.31) | | 4(1.67) | 3(1.02) |  |  |  |
| ASI scale of insomnia |  | |  |  | 0.0150 | 0.45(0.26,0.81) | 0.0070 |
| No insomnia | 464(86.89) | | 220(92.05) | 244(82.71) |  |  |  |
| Mild insomnia | 52(9.74) | | 13(5.44) | 39(13.22) |  |  |  |
| Moderate insomnia | 15(2.81) | | 5(2.09) | 10(3.39) |  |  |  |
| Severe insomnia | 3(0.56) | | 1(0.42) | 2(0.68) |  |  |  |
| PTSD |  | |  |  | 0.0325 | 3.24(1.22,8.64) | 0.0186 |
| Negative | 509(95.32) | | 233(97.49) | 276(93.56) |  |  |  |
| Positive | 25(4.68) | | 6(2.51) | 19(6.44) |  |  |  |

Data are n(%) or median (IQR). *OR or β(95%CI) obtained by logistic regression, rank logistic regression and linear regression, adjusted for age, comorbidities, length of hospital stay, corticosteroid, 5: admitted to hospital, requiring HFNC or non-IMV or both, 6: admitted to hospital, requiring ECMO or IMV or both. mMRC=modified British medical research council. mMRC=modified British medical research council. PHQ=patient health questionnaire. GAD=generalized anxiety disorder. ASI=arabic scale of insomnia. PTSD=posttraumatic stress disorder.

# Table S3. Health-related quality of life, PCFS scales and extrapulmonary organ function at 15-month follow-up according to sex

|  | Total  (n=534) | | Male  (n=239) | | Female  (n=295) | | p | | OR or β(95%CI)* | | P for regression | |
| --- | --- | --- | --- | --- | --- | --- | --- | --- | --- | --- | --- | --- |
| Events in one-year after discharge | |  | |  | |  | |  | |  | |  |
| Non-fatal myocardial infarction or non-fatal stroke | | 7(1.31) | | 2(0.84) | | 5(1.69) | | 0.6282 | | 2.16(0.39,11.81) | | 0.3756 |
| Heart failure hospitalization | | 5(0.94) | | 1(0.42) | | 4(1.36) | | 0.5050 | | 5.19(0.53,50.43) | | 0.1558 |
| Arterial revascularization therapy | | 11(2.06) | | 8(3.35) | | 3(1.02) | | 0.1144 | | 0.30(0.08,1.18) | | 0.0843 |
| New-onset venous thrombotic disease | | 9(1.69) | | 3(1.26) | | 6(2.03) | | 0.7211 | | 1.31(0.31,5.55) | | 0.7099 |
| Exacerbation of renal disease requires dialysis or kidney transplantation | | 1(0.19) | | 0(0.00) | | 1(0.34) | | 1.0000 | | 7353.0(0.00,22E108) | | 0.9427 |
| New-onset diabetes | | 19(3.56) | | 7(2.93) | | 12(4.07) | | 0.4799 | | 1.63(0.61,4.36) | | 0.3285 |
| New-onset AITD | | 10(1.87) | | 1(0.42) | | 9(3.05) | | 0.0561 | | 6.93(0.86,56.01) | | 0.0694 |
| New-onset neuropsychiatric disease | | 3(0.56) | | 1(0.42) | | 2(0.68) | | 1.0000 | | 1.98(0.16,23.89) | | 0.5914 |
| New-onset cancer | | 4(0.75) | | 3(1.26) | | 1(0.34) | | 0.4738 | | 0.13(0.01,1.61) | | 0.1120 |
| EQ-5D-5L questionnaire | |  | |  | |  | |  | |  | |  |
| Mobility: problems with walking | | 102(19.10) | | 45(18.83) | | 57(19.32) | | 0.8853 | | 1.22(0.74,2.00) | | 0.4361 |
| Personal care: problems with washing or dishing | | 70(13.11) | | 34(14.23) | | 36(12.20) | | 0.4911 | | 1.01(0.57,1.79) | | 0.9663 |
| Usual activity: problems with usual activity | | 85(15.92) | | 38(15.90) | | 47(15.93) | | 0.9918 | | 1.18(0.70,1.98) | | 0.5397 |
| Pain or discomfort | | 102(19.10) | | 45(18.83) | | 57(19.32) | | 0.8853 | | 1.30(0.80,2.11) | | 0.2946 |
| Anxiety or depression | | 111(20.79) | | 42(17.57) | | 69(23.39) | | 0.0995 | | 1.81(1.13,2.90) | | 0.0132 |
| Quality of life | | 85.50(80.00,90.00) | | 89.00(80.00,95.00) | | 85.00(78.00,90.00) | | 0.0680 | | -1.33(-4.37,1.71) | | 0.3920 |
| PCSF scale | |  | |  | |  | | 0.4312 | | 0.89(0.62,1.29) | | 0.5485 |
| F0 | | 348(65.17) | | 157(65.69) | | 191(64.75) | |  | |  | |  |
| F1 | | 13(2.43) | | 5(2.09) | | 8(2.71) | |  | |  | |  |
| F2 | | 13(2.43) | | 3(1.26) | | 10(3.39) | |  | |  | |  |
| F3 | | 95(17.79) | | 47(19.67) | | 48(16.27) | |  | |  | |  |
| F4 | | 65(12.17) | | 27(11.30) | | 38(12.88) | |  | |  | |  |

Data are n(%). *OR or β(95%CI) obtained by logistic regression, rank logistic regression and linear regression, adjusted for age, comorbidities, length of hospital stay, corticosteroid, 5: admitted to hospital, requiring HFNC or non-IMV or both, 6: admitted to hospital, requiring ECMO or IMV or both. mMRC=modified British medical research council. AITD=autoimmune thyroid disease. EQ-5D-5L=EuroQol 5-Dimension Questionnaire 5-level version. PCSF=post COVID-19 functional status.

# Table S4. Change of PTSD outcomes among COVID-19 patients between 3-month and 15-month follow-up

|  | 3 month  (n=325) | 15 month  (n=325) | P value |
| --- | --- | --- | --- |
| PTSD items |  |  |  |
| Re-experiencing | 77 (23.4) | 15 (4.6) | < 0.001 |
| Avoidance | 80 (24.3) | 28 (8.5) | < 0.001 |
| Hyperarousal | 83 (25.2) | 20 (6.1) | < 0.001 |
| Numbing | 90 (27.4) | 21 (6.4) | < 0.001 |
| Positive in PTSD | 76 (23.1) | 14 (4.3) | < 0.001 |

PTSD=posttraumatic stress disorder

# Table S5. Characteristics of COVID-19 patients with and without complete follow-up.

|  | Follow-up  (n = 525) | Lost  (n = 999) | Total  (n = 1524) | *P* value |
| --- | --- | --- | --- | --- |
| Age, year |  |  |  |  |
| Median(IQR) | 61 (51, 69) | 59 (49, 68) | 60 (50, 68) | 0.186 |
| Gender |  |  |  | 0.0436 |
| Male | 234 | 389 | 623 |  |
| Female | 290 | 601 | 891 |  |
| Consciousness disorder during hospitalization |  |  |  | 0.9202 |
| Yes | 6 | 12 | 18 |  |
| No | 519 | 987 | 1506 |  |
| Mental disorder history |  |  |  | 0.8687 |
| Yes | 16 | 32 | 48 |  |
| No | 509 | 967 | 1476 |  |
| Dementia |  |  |  | 0.3190 |
| Yes | 2 | 10 | 12 |  |
| No | 523 | 989 | 1512 |  |
| Cerebral vascular diseases history |  |  |  | 0.6140 |
| Yes | 12 | 19 | 31 |  |
| No | 513 | 980 | 1493 |  |

# COVID-19 patients 15-month follow-up questionnaire

1. Demographic data
2. Number:
3. Name:
4. Sex:
5. The post-COVID-19 Functional Status Scal (PCFS)[^[[1]](#endnote-1)^]
6. Can you live alone without the help of others? (For example, able to eat independently, take a walk, go to the toilet, manage daily personal hygiene, such as brushing teeth, bathing, dressing, etc.)

If not, Grade 4 (severe functional limitation)

If so, go to the question below.

1. Are you unable to complete certain tasks/activities independently in your daily life? (E.g. housework, daily exercise, etc.)

If yes, Grade 3 (moderate functional limitation)

If not, go to the question below.

1. Do you have certain symptoms, pain, depression or anxiety and other physical discomforts?

If not, Grade 0 (no functional limitation)

If yes, go to the question below.

1. Do you need to reduce work/activity time due to the aforementioned physical discomfort?

If yes, Grade 2 (mild functional limitation)

If no, Grade 1 (minimal functional limitation)

1. Current residual symptoms
2. Fever: yes/no, if yes, go to the following questions

fever type: continuous fever/intermittent fever (interval>24 hours)

Highest body temperature:＜37.5℃/37.5-38℃/≥38℃

1. Cough: yes/no, if yes, go to the following questions

Cough nature: dry cough/phlegm cough, such as phlegm cough, turn to the following questions

The nature of expectoration: white sputum/yellow sputum/blood sputum

1. Dyspnea: yes/no, if yes, determine Dyspnea level using the Modified Medical Research Council(mMRC) scale

Level 0 (Shortness of breath only during strenuous activity)

Level 1 (shortness of breath when walking fast or walking uphill)

Level 2 (walking on flat ground is slower than people of the same age or needs to stop and rest)

Level 3 (rest is required to walk 100m or several minutes on level ground)

Level 4 (difficulty breathing when unable to leave home or wearing or taking off clothes)

1. Fatigue: Yes/No
2. Memory loss: yes/no
3. Sleep disorders: yes/no
4. Hair loss: yes/no
5. Pharyngeal discomfort: yes/no
6. Olfactory disorders: yes/no
7. Taste disturbance: yes/no
8. Loss of appetite: yes/no
9. Nausea and vomiting: yes/no
10. Diarrhea: Yes/No
11. Palpitations: Yes/No
12. Chest tightness: yes/no
13. Chest pain: yes/no
14. Muscle pain: yes/no
15. Joint pain: yes/no
16. Headache: yes/no
17. Dizziness: yes/no
18. Skin rash: yes/no
19. Whether the following incident happened within 1 year after discharge from hospital
20. Death: yes/no, if yes, please explain the cause of death ()
21. Occurrence of non-fatal myocardial infarction and stroke: yes/no
22. Hospitalization due to heart failure: yes/no
23. Hospitalized due to diagnosis of coronary heart disease, carotid artery, lower extremity artery stenosis, balloon dilation, stent implantation and other arterial revascularization treatments: yes/no
24. New venous thrombosis events, including pulmonary embolism, deep vein thrombosis of the lower extremities: yes/no
25. Existing kidney disease is aggravated and requires dialysis or kidney transplantation: Yes/No
26. New-onset diabetes, including fasting blood glucose>7.0mmol/L: yes/no
27. New thyroid diseases, including hyperthyroidism, hypothyroidism, subacute thyroiditis, Hashimoto's thyroiditis (except thyroid nodules): yes/no
28. Diagnosed neuropsychiatric disease: yes/no, if yes, switch to the following options (dementia/Parkinson's disease/epilepsy/Guillain-Barre syndrome/schizophrenia/other, please specify)
29. Diagnosed cancer: yes/no, if yes, please specify ()
30. Quality of Life Questionnaire (EQ-5D-5L Questionnaire)[^[[2]](#endnote-2)^]
31. Walking ability
    (0-Can't walk; 1-Difficulty walking; 2-Moderate difficulty; 3-Mild difficulty; 4-No difficulty)
32. Personal care ability
    (0-cannot dress and bathe by yourself; 1-difficulty dressing and bathing by yourself; 2-moderate difficulty; 3-mild difficulty; 4-no difficulty)Daily sports ability, including work, study, housework, leisure sports
33. (0-Cannot exercise; 1-Daily exercise difficulty; 2-Moderate difficulty; 3-Mild difficulty; 4-No difficulty)
34. Pain/discomfort
    (0-very severe; 1-heavy; 2-moderate; 3-mild; 4-no pain/discomfort)
35. Depression/anxiety
    (0-very severe; 1-heavy; 2-moderate; 3-mild; 4-no depression/anxiety)
36. If you were to rate today's quality of life with a full score of 100, how many points would you rate?
37. Please choose according to your feeling frequency in the past week: (phq9 depression scale)[^[[3]](#endnote-3)^]
38. Lack of motivation or interest in doing things
    (0-not at all, 1-a few days, 2-more than half of the time, 3-almost every day)
39. Feeling down, depressed, or desperate
    (0-not at all, 1-a few days, 2-more than half of the time, 3-almost every day)
40. Difficulty falling asleep, restless sleep or more sleep
    (0-not at all, 1-a few days, 2-more than half of the time, 3-almost every day)
41. Feeling tired or not energetic
    (0-not at all, 1-a few days, 2-more than half of the time, 3-almost every day)
42. Loss of appetite or eating too much
    (0-not at all, 1-a few days, 2-more than half of the time, 3-almost every day)
43. Feel that you are bad or that you are a failure, or let yourself or your family down
    (0-not at all, 1-a few days, 2-more than half of the time, 3-almost every day)
44. Difficulty focusing on things, such as reading newspapers or watching TV
    (0-not at all, 1-a few days, 2-more than half of the time, 3-almost every day)
45. The movement or speaking speed is so slow that others have noticed it? Or just the opposite-irritability or fidgeting, moving around is better than usual
    (0-not at all, 1-a few days, 2-more than half of the time, 3-almost every day)
46. Thoughts that it is better to die or hurt yourself in some way
    (0-not at all, 1-a few days, 2-more than half of the time, 3-almost every day)
47. In the past two weeks, how often did the following symptoms appear in your life? (GAD-7 Anxiety Scale)[^[[4]](#endnote-4)^]
48. Feeling nervous, anxious or anxious
    (0-not at all, 1-a few days, 2-more than half of the time, 3-almost every day)
49. Unable to stop or control worry
    (0-not at all, 1-a few days, 2-more than half of the time, 3-almost every day)
50. Too much worry about all kinds of things
    (0-not at all, 1-a few days, 2-more than half of the time, 3-almost every day)
51. It's hard to relax
    (0-not at all, 1-a few days, 2-more than half of the time, 3-almost every day)
52. Unable to sit still due to restlessness
    (0-not at all, 1-a few days, 2-more than half of the time, 3-almost every day)
53. Becomes easily upset or irritable
    (0-not at all, 1-a few days, 2-more than half of the time, 3-almost every day)
54. Feel that something terrible will happen and be afraid
    (0-not at all, 1-a few days, 2-more than half of the time, 3-almost every day)
55. Regarding your sleep status in the last month, please select or fill in the answer that best suits your actual situation: (ASI Sleep Scale)[^[[5]](#endnote-5)^]
56. Difficulty falling asleep
    (0-no or almost none; 1-slight or a little bit; 2-generally severe or moderate; 3-severe; 4-very severe)
57. It is easy to wake up at night
    (0-no or almost no; 1-slight or a little; 2-generally severe or moderate; 3-severe; 4-extremely severe)
58. Early awakening
    (0-no or almost none; 1- mild or a little bit; 2- generally severe or moderate; 3- severe; 4- extremely severe)
59. Your satisfaction with your sleep status in the past month
    (0-very satisfied; 1-satisfied; 2-fair; 3-unsatisfied; 4-very dissatisfied)
60. How do you think your sleep problems interfere with your daytime life? (Such as daytime fatigue, ability to handle daily tasks, concentration, memory and mood, etc.)
    (0-no effect at all; 1-occasionally; 2-slightly affected; 3-more influential; 4-very influential)
61. To what extent do you think sleep problems affect the quality of life?
    (0-No effect at all; 1-Occasionally affected; 2-Slightly affected; 3-Slightly affected; 4-Very influential)
62. How much are you anxious/worry about your current sleep problems?
    (0-no; 1-somewhat; 2-some; 3-somewhat worried; 4-very worried)
63. Total sleep time after discharge: hour/night
64. Whether to take sleeping medicine after discharge: Yes/No, if yes, please fill in the name of the medicine.
65. Please choose according to your feelings in the last month: (simple PTSD scale, PC-PTSD, primary care PTSD screen)[^[[6]](#endnote-6)^]
66. Repeatedly think of experiences related to new coronary pneumonia or repeatedly have nightmares about new coronary pneumonia: Yes/No
67. Efforts to avoid memories or related people, places, activities or scenes related to COVID-19: Yes/No
68. Always be vigilant, over-alert, or easily frightened: yes/no
69. Feeling numb or out of touch with others, daily activities or the surrounding environment: Yes/No
70. Feeling guilty or ashamed, or blaming yourself or others for what happened after COVID-19: Yes/No
71. I feel that someone discriminates against me because I have suffered from new coronary pneumonia: (1 Disagree at all, 4 Not sure, 7 Completely agree). From scale 1-7.
72. Questionnaire narrator: the patient/the patient’s family

1. [] Klok FA, Boon GJAM, Barco S, Endres M, Geelhoed JJM, Knauss S, Rezek SA, Spruit MA, Vehreschild J, Siegerink B. The Post-COVID-19 Functional Status scale: a tool to measure functional status over time after COVID-19. Eur Respir J. 2020 Jul 2;56(1):2001494. doi: 10.1183/13993003.01494-2020. PMID: 32398306; PMCID: PMC7236834. [↑](#endnote-ref-1)
2. [] Rabin R, de Charro F. EQ-5D: a measure of health status from the EuroQol Group. Ann Med. 2001 Jul;33(5):337-43. doi: 10.3109/07853890109002087. PMID: 11491192. [↑](#endnote-ref-2)
3. [] Kroenke K, Spitzer RL, Williams JB. The PHQ-9: validity of a brief depression severity measure. J Gen Intern Med. 2001 Sep;16(9):606-13. doi: 10.1046/j.1525-1497.2001.016009606.x. PMID: 11556941; PMCID: PMC1495268. [↑](#endnote-ref-3)
4. [] Plummer F, Manea L, Trepel D, McMillan D. Screening for anxiety disorders with the GAD-7 and GAD-2: a systematic review and diagnostic metaanalysis. Gen Hosp Psychiatry. 2016 Mar-Apr;39:24-31. doi: 10.1016/j.genhosppsych.2015.11.005. Epub 2015 Nov 18. PMID: 26719105. [↑](#endnote-ref-4)
5. [] [HYERYEON YI](https://onlinelibrary.wiley.com/action/doSearch?ContribAuthorStored=YI,+HYERYEON), [KYUNGRIM SHIN](https://onlinelibrary.wiley.com/action/doSearch?ContribAuthorStored=SHIN,+KYUNGRIM), [CHOL SHIN](https://onlinelibrary.wiley.com/action/doSearch?ContribAuthorStored=SHIN,+CHOL). Development of the Sleep Quality Scale. Sleep Hypn. 2008;10(1):3-10. [doi: 1365-2869.2006.00544.x](https://doi.org/10.1111/j.1365-2869.2006.00544.x) [↑](#endnote-ref-5)
6. [] Prins A, Bovin MJ, Smolenski DJ, Marx BP, Kimerling R, Jenkins-Guarnieri MA, Kaloupek DG, Schnurr PP, Kaiser AP, Leyva YE, Tiet QQ. The Primary Care PTSD Screen for DSM-5 (PC-PTSD-5): Development and Evaluation Within a Veteran Primary Care Sample. J Gen Intern Med. 2016 Oct;31(10):1206-11. doi: 10.1007/s11606-016-3703-5. Epub 2016 May 11. PMID: 27170304; PMCID: PMC5023594. [↑](#endnote-ref-6)
